# Residual malignant and normal plasma cells shortly after high dose melphalan and stem cell transplantation. Highlight of a putative therapeutic window in Multiple Myeloma?

**DOI:** 10.18632/oncotarget.650

**Published:** 2012-10-25

**Authors:** Anouk Caraux, Laure Vincent, Salahedine Bouhya, Philippe Quittet, Jérôme Moreaux, Guilhem Requirand, Jean-Luc Veyrune, Gaëlle Olivier, Guillaume Cartron, Jean-François Rossi, Bernard Klein

**Affiliations:** ^1^ INSERM, U1040, Montpellier, France; ^2^ CHU Montpellier St Eloi, Institute of Research in Biotherapy, France; ^3^ CHU Montpellier St Eloi, Service d'Hématologie et Oncologie Médicale, France; ^4^ CHU Montpellier St Eloi, Cell Therapy Unit, France; ^5^ Université Montpellier 1, France

**Keywords:** Multiple Myeloma, Plasma cells, Minimal residual disease, Human

## Abstract

Multiple Myeloma (MM) is an incurable malignant plasma cell disorder. We have evaluated the counts of Multiple Myeloma Cells (MMCs) and normal plasma cells (N-PCs), seven days after high-dose melphalan (HDM) and autologous stem transplantation (ASCT). Two third of patients had detectable minimal residual disease (MRD^+^) (71.7 MMCs/μL) after induction treatment with dexamethasone and proteasome inhibitor. MMC counts were reduced by 92% (P ≤ .05) but not eradicated 7 days after HDM+ASCT. Post-HDM+ASCT MMCs were viable and bathed in a burst of MMC growth factors, linked with post-HDM aplasia. In one third of patients (MRD^−^ patients), MMCs were not detectable after induction treatment and remained undetectable after HDM+ASCT. Major difference between MRD^−^ and MRD^+^ patients is that N-PC counts were increased 3 fold (P < .05) by HDM+ASCT in MRD^−^ patients, but were unaffected in MRD^+^ patients. Possible explanation could be that clearance of MMCs in MRD^−^ patients makes more niches available for N-PCs. Thus, MMCs are not fully eradicated shortly after HDM, are bathed in high concentrations of MMC growth factors in an almost desert BM, are viable in short-term culture, which suggests providing additional therapies shortly after HDM to kill resistant MMCs before full repair of lesions.

## INTRODUCTION

Multiple myeloma (MM) is characterized by the accumulation of clonal malignant plasma cells (PCs), primarily in the bone marrow (BM). It affects every year 86000 new patients throughout the world.[1] MM is still an incurable disease with a median survival in excess of 5 years.[2] For patients below 65-70 years, first line treatment includes 4-6 cycles of induction therapy based on novel agents (proteasome inhibitor or immunomodulatory drugs), high dose dexamethasone (D) and DNA targeting drugs, followed by intensification treatment with high dose melphalan (HDM) supported by autologous stem cell transplantation (ASCT). Autologous hematopoietic stem cells (HSCs) are collected after 3 to 4 cycles of induction therapy, HSC being mobilized into peripheral blood (PB) using granulocyte colony-stimulating factor (G-CSF) alone or in combination with cyclophosphamide. The association of immunomodulatory drugs (Thalidomide® or Revlimid®) to proteasome inhibitor (Bortezomib, B) and high doses dexamethasone improves the rates of complete responses after induction and intensification treatment.[3] The major therapeutic questions addressed by large ongoing clinical trials concern the remaining role of HDM and ASCT versus novel agent combinations alone without intensification and the place for consolidation/maintenance therapies.

In parallel to large randomized clinical trials, it could be useful to carefully evaluate the effect of a reference treatment option on malignant plasma cells (multiple myeloma cells, MMCs) as well as on their normal plasma cell (N-PC) counterpart *in vivo*. The use of multiparameter flow cytometry (MFC) makes it possible to discriminate and count MMCs and N-PCs in the BM or PB of patients.[4] The opportunity to acquire and compute large numbers of events (5 × 10^6^ cells) allows the detection of 1 MMC or N-PC in 10^5^ cells.[5] Using a similar approach, Paiva *et al*. showed that minimal residual disease (MRD) assessed 3 months after HDM and ASCT, is the most powerful independent prognosis factor for event free and overall survival.[6] More recently, this group showed that negative MFC immunophenotypic response is an earlier marker than immunofixation to predict complete response, at least with a 10^−5^ sensitivity.[7]

In this study, we address the question of the effects of HDM and ASCT on MMCs and N-PCs. For two third of the patients, MMCs were detected in the BM after BD induction treatment. HDM and ASCT reduced but did not eradicate these MMCs. These post-HDM MMCs are viable and bathed in a burst of MMC growth factors, likely induced by post HDM aplasia. This suggests providing additional hits shortly after HDM to kill these resistant MMCs before full repair of lesions.

## RESULTS

### Detection of viable myeloma cells 7 days after high dose melphalan and autologous stem cell transplantation in patients with residual multiple myeloma cells one day before melphalan

After induction treatment with 4-6 courses of BD, MMCs could be detected in BM samples of 18/27 (67%) patients the day before HDM infusion. These 18 patients are termed patients with positive MRD (MRD^+^). The median MMC count was 71.7 MMCs/μL (range 0.4-285.5 MMCs/μL) (Figure 2A and Table 2). For 17 of the 18 MRD^+^ patients, MMCs could still be detected in the BM 7 days after HDM and ASCT, however with a 92% reduction in median MMC counts (5.5 MMCs/μL versus 71.7 MMCs/μL, *P* ≤ .001) (Table 2). Post-HDM residual MMCs were viable cells, since they could survive for 6 days when cultured *in vitro* (Figure 2B). MMCs were evaluated in the grafted stem cell products of 16 of the 18 MRD^+^ patients. For 11 of these 16 MRD^+^ patients, the thawed stem cell products grafted to patients contained MMCs (Table 3). The median MMC count was 0.03 × 10^6^ cells/kg, range 0-1.2 × 10^6^ cells/kg, 100 fold lower than the median count of grafted CD34 stem cells (Table 3). BM could be collected 3 months after HDM for 12 of the 18 MRD^+^ patients. MMCs were detected in 9 of these 12 samples with a median MMC count of 2.4 cells/μL at 3 months (Table 2).

**Figure 1:**
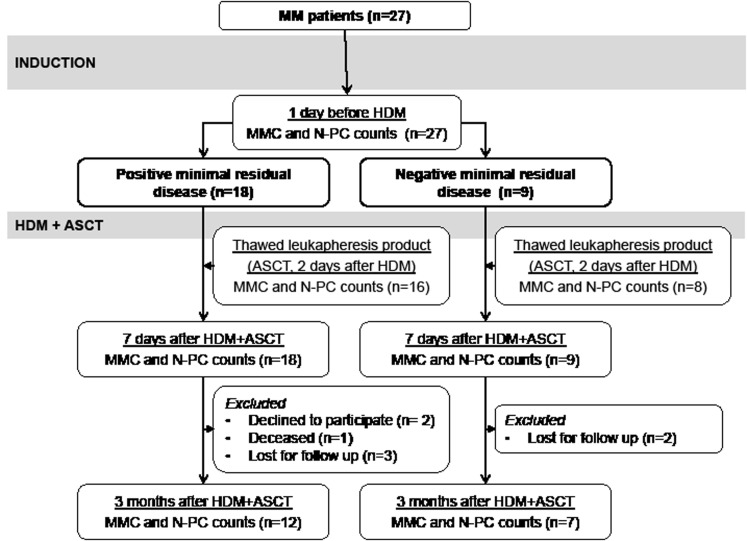
CONSORT diagram of patients with previously-untreated multiple myeloma in Montpellier University Hospital, showing number of patients, treatments delivered, and outcome MRD, minimal residual disease; HDM, high dose melphalan; ASCT, autologous hematopoietic stem cells transplantation; MFC, multiparameter flow cytometry.

**Figure 2:**
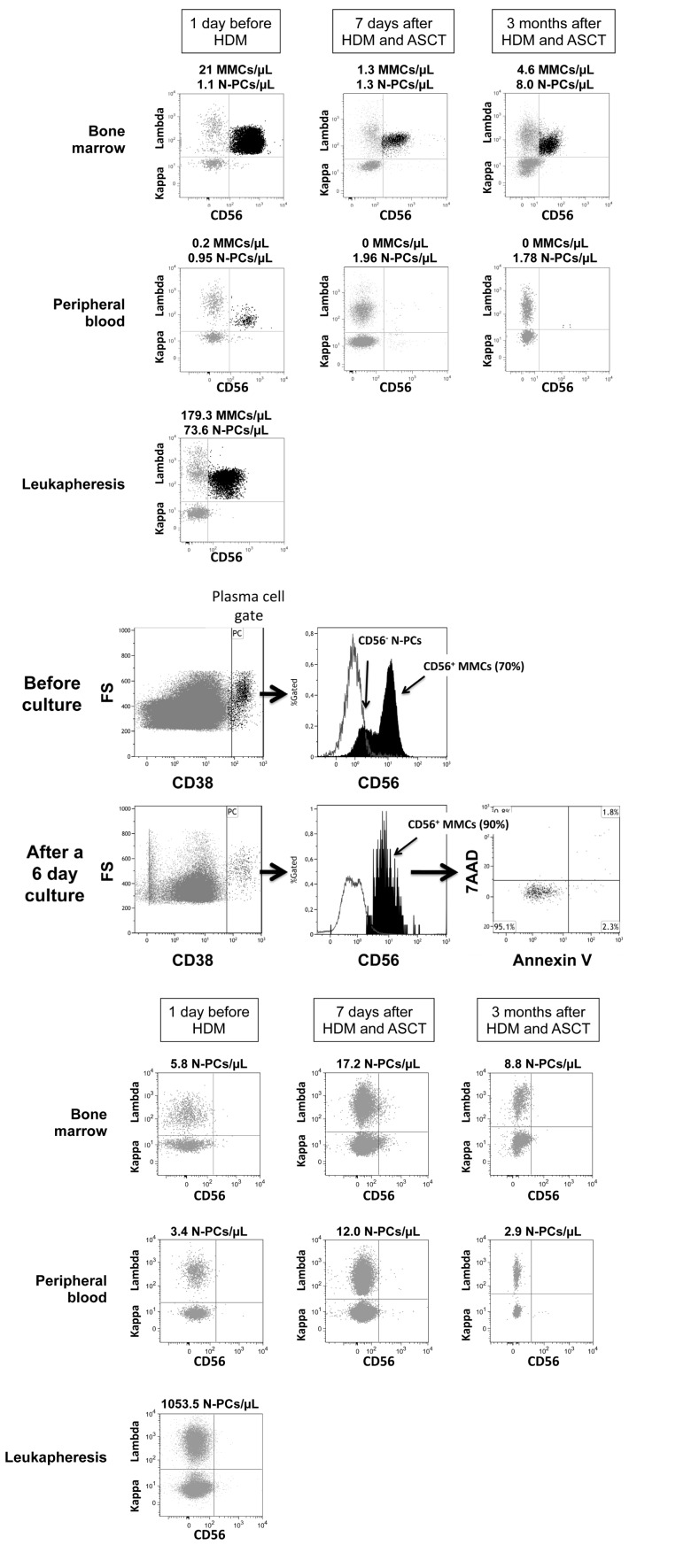
Assessment of Multiple Myeloma Cells and Normal Plasma cells in representative patients with Multiple Myeloma before and after high dose melphalan Using 7 color-multiparameter flow cytometry, multiple myeloma cells (MMCs) and normal plasma cells (N-PCs) were assessed in bone marrow or peripheral blood samples of patients after induction treatment (1 day before high dose melphalan, HDM), 7 days after HDM and autologous hematopoietic stem cell transplantation (ASCT), 3 months after HDM. MMCs and N-PCs were also measured in the thawed stem cell leukapheresis product grafted to the patients. (A) MMC and N-PC evaluation in a representative patient with positive residual disease after induction treatment (MRD^+^). MMCs were identified on the basis of aberrant CD56 expression and monoclonal lambda light chain expression. Data are the dotplots of CD56 and Lambda light chain expressions. MMCs were undetectable 7 days and 3 months after HDM+ASCT in the peripheral blood. (B) Bone marrow cells from one representative MRD^+^ patient out of 3 were harvested 7 days after HDM+ASCT and cultured for 6 days with 2 ng/mL of IL-6. Before culture, bone marrow cells contained CD38^high^ CD56^+^ MMCs (70% of CD38^high^ cells) and CD38^high^CD56^−^ N-PCs. After a 6-day, the CD38^high^ CD56^+^ MMCs were viable, being Annexin V^−^ 7AAD^−^. (C) N-PC evaluation and lack of MMCs in a representative patient with negative residual disease after induction treatment. MMCs of this patient aberrantly expressed CD56 and CD200 at diagnosis. After induction treatment, only N-PCs could be detected in the bone marrow, peripheral blood and leukapheresis product.

**Table 1:** Patient demographics and baseline characteristics

	n=27
Male / female; number (%)	14 / 13 (52 / 48)
Age, years; median (range)	59 (40-67)
Myeloma subtype, (%)	
IgG	26
IgA	26
Light chain	33
Non secretory	7
Other	7
Durie-Salmon disease stage I / II / III, (%)	22 / 19 / 59
International Staging System disease stage I / II / III, (%)	61 / 17 / 22
β2-microglobulin, (mg/L); median (range)	3 (1.6 - 44.5)
Hemoglobin, g/L; median (range)	11 (5.5 - 15.4)
Creatinine, μmol/L; median (range)	93 (45 - 336)
Calcium, mmol/L; median (range)	2.5 (2.3 - 4.4)
Albumin, g/L; median (range)	40 (30 - 53)
Plasma cells by morphology, %	36 (10 - 72)
Malignant plasma cell phenotype, cases by 7-color flow cytometry, (%)	
CD19−	92
CD20+	17
CD27−	67
CD45−	88
CD56+	67
CD117+	25
CD200+	71

**Table 2:** Multiple myeloma cell and normal plasma cell counts in bone marrow and peripheral blood samples one day before and 7 days after high dose melphalan and autologous stem cell transplantation

	One day before HDM	7 days after HDM and ASCT	3 months after HDM and ASCT
**Bone marrow**			
**Patients with positive MRD the day before HDM**			
Number of patients evaluated	18	18/18	12/18
Bone marrow cell count (106 cells/mL)	8.4 (3.7-27.0)	0.3 (0.1-3.6)	9.8 (3.9-20.6)
Detection of MMCs	Detected in 18/18 samples	Detected in 17/18 samples	Detected in 9/12 samples
MMC count (cells/μL)	71.7 (0.4-285.5)	5.5 (0-76.9)	2.4 (0-68.3)
N-PC count (cells/μL)	4.3 (0.7-17.0)	3.3 (0.3-32.2)	6.1 (0.3-21.5)
B-lymphocyte count (cells/μL)	22.7 (4.8-126.6)	0.9 (0-14.2)	184.4 (58.5-368.7)
**Patients with negative MRD the day before HDM**			
Number of patients evaluated	9	9/9	7/9
Bone marrow cell count (106 cells/mL)	9.4 (6.0-28.9)	0.9 (0.2-2.0)	8.3 (6.5-26.5)
Detection of MMCs	Undetectable	Undetectable in 9/9 samples	Undetectable in 6/7 samples
N-PC count (cells/μL)	5.8 (1.4-32.0)	17.2 (0.4-199.0)	6.5 (0.9-26.8)
B-lymphocyte count (cells/μL)	58.3 (11.3-324.9)	1.8 (0.3-2.5)	250.8 (18.2-511.5)
**Peripheral blood**			
**Patients with positive MRD the day before HDM**			
Number of patients evaluated	17/18	14/18	10/18
Peripheral blood cell count (106 cells/mL)	4.7 (3.3-11.1)	0.1 (0.1-0.6)	5.2 (2.7-10.1)
Detection of MMCs	Detected in 12/18 samples	Detected in 6/14 samples	Detected in 0/10 samples
MMC count (cells/μL)	0.1 (0-0.4)	0.002 (0-0.2)	0
N-PC count (cells/μL)	0.9 (0.1-4.2)	0.9 (0.2-4.3)	0.5 (0-8.5)
B-lymphocyte count (cells/μL)	24.4 (3.1-94.4)	0.2 (0.04-2.5)	129.2 (40.5-389.3)
**Patients with negative MRD the day before HDM**			
Number of patients evaluated	9	9/9	5/9
Peripheral blood cell count (106 cells/mL)	4.6 (3.1-7.4)	0.3 (0.1-1.6)	4.0 (2.6-5.1)
Detection of MMCs	Undetectable in 9/9 samples	Undetectable in 9/9 samples	Undetectable in 5/5 samples
N-PC count (cells/μL)	1.8 (0.5-3.4)	3.6 (0.2-12.8)	0.8 (0.03-3.8)
B-lymphocyte count (cells/μL)	26.1 (7.3-443.7)	0.3 (0.1-0.7)	123.4 (76.4-265.0)

Results expressed as median (range) ; Autologous stem cell transplantation (ASCT) ; High dose melphalan (HDM) ; Minimal residual disease (MRD) ; Normal plasma cell (N-PC) ; Multiple myeloma cell (MMC)

**Table 3:** Counts of Multiple myeloma cells and normal plasma cells in leukapheresis products

	Leukapharesis product
**Patients with positive MRD the day before HDM**	
Patients' Number	16
Leukapheresis cell count (106 cells/mL)	160.3 (54.2-226.6)
Detection of MMCs	11/16
MMC count (106 cells/kg)	0.03 (0-1.2)
N-PC count (106 cells/kg)	0.5 (0.1-6.9)
B-lymphocyte count (cells/μL)	2387.2 (86.5-10723.0)
CD34+ cell count (106 cells/kg)	2.9 (1.7-5.0)
**Patients with negative MRD the day before HDM**	
Patients' Number	8
Leukapheresis cell count (106 cells/mL)	115.3 (68.7-192.1)
Detection of MMCs	0/8
N-PC count (106 cells/kg)	1.6* (0.4-22.4)
B-lymphocyte count (cells/μL)	2077.3 (99.5-5206.9)
CD34+ cell count (106 cells/kg)	2.8 (2.1-17.2)
Results expressed as median (range)	

High dose melphalan (HDM) ; Minimal residual disease (MRD) ; Normal plasma cell (N-PC) ; Multiple myeloma plasma cell (MMC)

* Significantly increased compared to N-PC counts in leukapheresis products of MRD+ patients

### No detection of multiple myeloma cells 7 days after high dose melphalan and autologous stem cell transplantation in patients with no residual myeloma cells the day before melphalan

Thirty-three percent of the patients (9/27) had no detectable MMCs (< 1 MMC/10^5^ cells) in BM samples the day before HDM infusion (Table 2). These patients are named MRD^−^ patients. Representative cytometry data of one patient are shown in Figure 2C. No MMCs could be detected either in the BM of these 9 MRD^−^ patients harvested 7 days after HDM and ASCT. No MMCs were detected in the thawed stem cell products of these MRD^−^ patients (Table 3). Seven of the 9 MRD^−^ patients could be evaluated 3 months after HDM. Only in one patient, a low count of BM MMCs was detected (1.3 MMCs/μL).

### The count of peripheral blood multiple myeloma cell is not a sensitive indicator of minimal residual disease

PB MMCs were undetectable in 6 of the 18 patients with detectable BM MMCs one day before HDM and in 8 of 14 patients with detectable BM MMCs 7 days after HDM and ASCT. Moreover 3 months after HDM, none of the 11 patients with detectable BM MMCs had detectable circulating MMCs (Table 2). No patients without detectable MMCs in the BM one day before HDM had detectable PB MMCs.

### Increase in normal plasma cell counts early after high dose melphalan and stem cell transplantation in MRD- patients

N-PCs were detected in all BM or PB samples harvested 1 day before HDM, 7 days after HDM+ASCT, and 3 months after HDM (Table 2 and Supplemental Figure S2). In MRD^+^ patients, the median N-PC count one day before HDM was 4.3 cells/μL and was not significantly different from those measured 7 days and 3 months after HDM+ASCT (Table 2). In MRD^−^ patients, the median N-PC count one day before HDM was 5.8 cells/μL, and was not significantly different from that in MRD^+^ patients (Table 2). However 7 days after HDM+ASCT, the median N-PC count in the BM was 3-fold higher than before HDM (17.2 N-PC/μL versus 5.8 N-PC/μL, *P* = .02). It was 5.2 fold higher in MRD^−^ patients compared to MRD^+^ patients (17.2 N-PC/μL versus 3.3 N-PC/μL, *P* = .02). The same holds true for peripheral blood. In MRD^−^ patients, N-PC counts after HDM were significantly increased 2 fold compared to those before N-PCs, whereas they were similar in MRD^+^ patients (Table 2 and Supplemental Figure S2). Interestingly, the stem cell products collected from MRD^−^ patients and grafted to these patients contained a significant 3-fold higher N-PC count (*P* < .05, 1.6 × 10^6^ viable N-PCs/kg) than those from MRD^+^ patients (0.5 × 10^6^ N-PCs/kg). In MRD^−^ patients, the median N-PC count in the thawed stem cells product grafted to patients was close to the median CD34 stem cell count (1.6 × 10^6^ N-PCs /kg versus 2.8 × 10^6^ CD34^+^ cells/kg) (Table 3). Three months after HDM, the median N-PC count in MRD^−^ patients dropped to 6.5 cells/μL and was similar to that in MRD^+^ patients (6.1 cells/μL). Of note, B lymphocyte counts (see Supplemental Figure S1) were similar between MRD^−^ and MRD^+^ patients 7 days after HDM and ASCT, in thawed leukapheresis products as well as before and 3 months after HDM (results not shown).

### Burst of myeloma cell growth factor production 7 days after high dose melphalan and autologous stem cell transplantation

**Figure 3:**
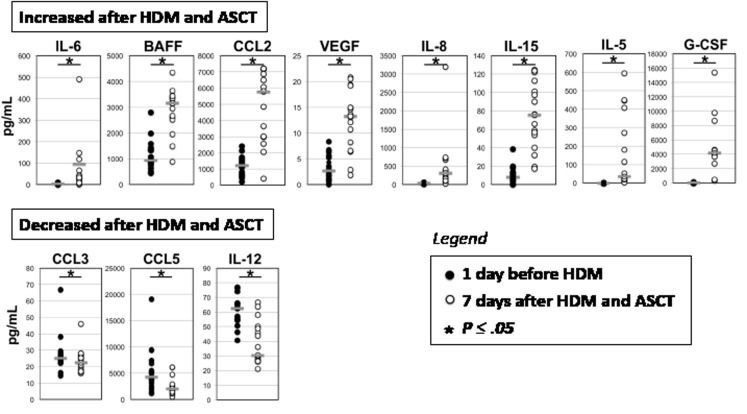
Cell communication proteins in bone marrow plasma before and 7 days after high dose melphalan (HDM) and autologous stem cell transplantation (ASCT) Paired bone marrow plasmas from 17 patients were collected 1 day before and 7 days after HDM+ASCT (respectively black circles and open circles) and assayed for cell communication proteins using Luminex methodology or ELISA. Data are the concentrations of IL-6, BAFF, CCL2, VEGF, IL-8, IL-15, IL-5, G-CSF, CCL3, CCL5 and IL-12 medullary plasma concentrations measured for each of the 17 patients. Horizontal bars represent the median value. The * indicates that the data before and after HDM are significantly different (P ≤ 0.05) using a paired t-test (when normality test passed) or Wilcoxon signed rank test (when normality test failed).

Twenty-three cell communication proteins could be detected (above detection limits) in the BM plasma of MM patients using Luminex or ELISA methodologies. Only BAFF concentration was significantly different (*P* = .003) between MRD^−^ and MRD^+^ patients before HDM treatment. It was significantly 2 fold higher in MRD^−^ patients (1753.6 pg/mL) compared to MRD^+^ patients (910.3 pg/mL). Cell communication proteins were also investigated in paired BM plasma of 17 patients harvested 1 day before and 7 days after HDM and ASCT. Concentrations of IL-6, VEGF, BAFF, CCL2, IL-8, IL-15, and IL-5 significantly increased respectively 4.8, 6.7, 119.4, 8.8, 9.4, 9.2 and 4.9 fold (*P* < .05) 7 days after HDM and ASCT (Figure 3). On the other hand, the concentrations of CCL3, IL-12, and CCL5 decreased significantly 1.2, 2.1 and 2.1 fold respectively (*P* < .05). The concentrations of other 13 cell communication proteins, in particular of IGF1, IL-21, and APRIL, were not affected by HDM treatment (data not shown). The rise in G-CSF concentration observed 7 days after HDM+ASCT is hardly surprising since patients received pegylated G-CSF on day 5 after autologous stem cell graft (Figure 3).

## DISCUSSION

Several interesting observations were provided by the current study investigating the early effects of HDM+ASCT on MMCs and N-PCs. First, in 2 third of patients, residual MMCs could be detected in the BM after BD induction treatment in agreement with previously-reported response rate[2], and HDM reduced by 92% residual MMC counts but did not eradicate them. Residual post-HDM MMCs in the BM are viable cells and are bathed in high concentrations of MMC growth factors, particularly IL-6, BAFF, VEGF, and CCL2.[8] Given the very low counts of residual MMCs post HDM and ASCT, all BM sample had to be used to count MMCs and N-PCs and it was not possible to investigate whether these residual MMCs were in the cell cycle and underwent DNA repair, and this in comparison with their normal counterpart. But once flow cytometry methodologies to measure cell cycle and genomic instability will improve, this should be critical to investigate. An important point is the location and the survival niche of these post HDM residual MMCs. Primary MMCs are strongly dependent on the BM environment and purified MMCs dye within 3 days in *in vitro* culture once separated from their environment.[9] Niche cells supporting primary MMC[10-12] survival include endothelial cells[13], stromal cells[14-16], osteoblasts[17], osteoclasts[18-20], but also a large number of differentiated short-living hematopoietic cells: monocytes, myeloid cells[21] and megakaryocytes.[22] Shortly after HDM, short-living niche cells are cleared and the BM is almost desert. One can speculate that part of HDM effectiveness to reduce MMC load could be due to the clearance of short-living hematopoietic cells supporting MMC survival. The residual MMCs are likely bound to a few HDM resistant environment cells, at least endothelial cells, stromal cells, osteoblasts, and osteoclasts. Because of bleeding risk, it was not possible to perform bone biopsies early post HDM to identify niche cells binding resistant MMC and promoting their survival. The identification of these HDM resistant niche cells should be useful to design strategies targeting MMC/niche cell interactions for early post-HDM consolidation. In particular, it should be interesting to investigate whether residual MMCs are located far from the vessels, suggesting exposure to lower melphalan concentration, as documented for drug resistance in pancreatic cancer cells.[23] Promising strategies might be to inject anti-MMC monoclonal antibodies to kill post-HDM MMCs through complement dependent cytotoxicity or antibody dependent cell cytotoxicity (ADCC), providing these mAbs would not target and kill HSCs. Since there is a huge rise of activated monocytes during post ASCT hematopoietic recovery, this window could be optimal to promote ADCC to residual MMCs. Of note, MMCs express CS1/SLAMF7 [24-26], CD200 [27, 28], and CCR2 [19, 29] and mAbs to these proteins are already being investigated in clinical trials[30]. Anti-cytokine therapies could also be of interest to block residual MMC survival. In particular, our group has shown that anti-IL-6 mAb therapy is feasible throughout HDM and ASCT with no adverse effects regarding hematopoietic recovery.[31]

A second intriguing observation is that HDM and ASCT resulted in a 3-fold increase in N-PC counts in MRD^−^ patients, whereas N-PC counts are unaffected by HDM and ASCT in MRD^+^ patients. This increase was found both in BM and PB samples. N-PC counts were similar in the BM of MRD^−^ and MRD^+^ patients before HDM and 3 months after HDM and ASCT. This 3-fold increase in N-PC counts in MRD^−^ patients early after HDM and ASCT could be due to higher counts of grafted N-PCs. Indeed, the thawed leukapheresis products of MRD^−^ patients contained 3-fold more N-PCs (1.6 × 10^6^ N-PCs /kg) than those of MRD^+^ patients (0.5 × 10^6^ N-PCs /kg), whereas CD34 HSC counts (2.8 × 10^6^ CD34^+^ cells/kg versus 2.9 × 10^6^ CD34^+^ cells/kg) or B cell counts were similar. Given the known biology of N-PC, the current observation suggests that the ability of the immune system to produce N-PCs is more efficient in patients who will respond better to BD induction therapy. In healthy individuals, N-PCs are mostly long-surviving ones accounting for 10^9^ N-PCs in full body located in the BM and mucosa-associated lymphoid tissues.[32] New PCs are generated in the lymph nodes[33], exit into the PB (2 N-PCs/μL)[34], and have to home to BM or mucosa to find a niche providing survival signals.[32] Of note, some mature N-PCs may be induced to recirculate into PB in individuals vaccinated with tetanus toxoid likely due to a competition between newly generated PCs and old ones for a limited niche[35]. Similarly, mature N-PCs may be also mobilized into PB similar to HSCs in individuals receiving G-CSF.[36]

Providing niche cells available to support PC survival mainly regulate N-PC count[32], the increased N-PC count after high dose cyclophosphamide and G-CSF treatment or after HDM and ASCT in patients achieving negative MRD suggests the number of PC niche is increased in these patients. One can speculate that in MRD^−^ patients, BD induction treatment clear MMCs from a vast majority of PC niches making more niches available for N-PCs.[37] Alternatively, one can speculate that stromal cells from patients who will achieve negative MRD are more prompt to support N-PC survival, whereas those from MRD^+^ patients are more prompt to support MMC survival. In particular, we and others, have shown that BM stromal cells from patients with active MM have a different gene expression profiling than those from age-related healthy individuals.[14]

In conclusion, multicolor flow cytometry makes it possible in each patient to visualize the effects of a given drug combination on MMCs and also on their normal PC counterpart. This was applied here to HDM and ASCT treatment, which highlights a promising window to understand some mechanisms of drug resistance, and design new treatments to overcome it.

## DESIGN AND METHODS

### Patients, treatment, and response assessment

Twenty-seven consecutive MM patients from a single center (University Hospital, Montpellier, France) were included in this study (Figure 1). All patients had symptomatic untreated MM. Patients’ characteristics at diagnosis are shown in Table 1. Samples were collected after patients’ written informed consent in accordance with the Declaration of Helsinki and institutional research board approval from Montpellier University hospital (N° DC-2008-417). According to the French standard first line treatment, patients received an induction treatment of 4-6 cycles of BD followed by high-dose melphalan (HDM, 200 mg/m^2^) and ASCT. After the third BD cycle, patients received 10 μg/kg/day of G-CSF or 10 μg/kg/day of G-CSF plus 2 g/m^2^ cyclophosphamide in order to mobilize and collect HSCs. Response to therapy was assessed according to international criteria modified to include the category of near complete response (nCR: electrophoresis negative for M-protein, but immunofixation positive).[38, 39] Response assessment to BD induction (1 day before HDM) showed 48 % complete remission (CR) or nCR, 22 % of very good partial responses (VGPR) and 22 % of partial responses (PR). Three months after transplantation, 62% of patients were in CR/nCR, 21% in VGPR, and 8% in PR.

### Cell samples

PB and BM samples were harvested i) after induction treatment (i.e. 1 day before HDM, ii) 7 days after ASCT (*i.e.* 9 days after HDM), and iii) 3 months after HDM+ASCT. An aliquot of the thawed leukapheresis stem cell product grafted to the patient was also assessed. Erythrocytes were lysed with NH_4_Cl (0.7 mol/L) and leukocyte counts determined using ABX PENTRA 60 Analyzer (HORIBA ABX, Montpellier, France).

### Antibodies

Monoclonal antibodies (mAbs) conjugated to fluorescein isothiocyanate (FITC), phycoerythrin (PE), energy-coupled dye (ECD), PE-cyanin 5.5 (PE-Cy5·5), PE-Cy7, Pacific Blue, and allophycocyanin (APC), specific for human CD27 (clone L128), CD56 (N-CAM, clone B159), CD117 (clone 104D2), CD138 (clone MI15), lambda immunoglobulin light chain (Ig Lambda, clone JDC-12), and kappa immunoglobulin light chain (Ig Kappa, clone TB 28-2) were purchased from Becton/Dickinson (BDi) Biosciences (San Jose, CA). CD19 (clone J3-119), CD20 (clone B9E9), CD34 (clone 581), CD38 (clone LS198.4.3), CD45 (clones J33), and CD138 (clone B-A38) were obtained from Beckman Coulter (Fullerton, CA). CD200 (clone OX104) was purchased from eBiosciences (San Diego, CA).

### Multiparameter flow cytometry immunophenotyping

Multiparameter flow cytometry immunophenotyping was performed using a 7-color immunofluorescence technique as indicated.[34] Erythrocyte-lysed bone marrow, peripheral blood or leukapheresis samples were labeled with anti-CD19, CD20, CD38 and CD45 mAbs in all 7-color panels in association with either anti-CD138, CD27, CD56, CD117, CD200 or isotype control, namely CD138, CD27, CD56, CD117, CD200 or isotype control panels. Isotype control panel allowed evaluating levels of background fluorescence on plasma cell (PC) population.

Cells were then fixed and permeabilized with the Cytofix/Cytoperm kit (BDi Biosciences), and labeled with anti-Ig Kappa and anti-Ig Lambda mAbs. Data acquisition was performed with a Cyan flow cytometer (Beckman Coulter), driven by Summit 4.3 software (Beckman Coulter). A two-step acquisition procedure was adopted. In the first step, 5 × 10^6^ nucleated cells were acquired from the CD138 panel. This panel enabled the identification and counts of B-lymphocytes and PCs. The second step aimed at improving the sensitivity of PCs detection and at precisely assessing the counts of normal PCs (N-PCs) and multiple myeloma cells (MMCs). A broad “live-gate” was drawn to select cells with high CD38 expression (i.e. CD38^hi^ events). They were recorded and stored for the so-called CD27, CD56, CD117, CD200, and isotype control, 7 color panels as described.[6]

The Supplemental Figure S1 describes the multiparameter flow cytometry strategy used to differentiate B-lymphocytes, N-PCs and MMCs. Cell debris, platelets and doublets were excluded in a first step. B-lymphocytes were defined as [CD19^+^CD20^+^CD45^+^CD38^−/+^ and (Kappa^+^ or Lambda^+^)] cells and PCs as [CD38^high^ and (Kappa^+^ or Lambda^+^) and (not B lymphocytes)] cells. MMCs were identified on the basis of the monoclonal expression of Kappa or Lambda light chains together with the aberrant expression of one or several myeloma markers: expression of CD20 and/or CD56 and/or CD117 and/or CD200, lack of expression of CD19, and lack or weak expression of CD27 and/or CD45 (Supplemental Table S1).[4, 27, 28, 40] As MMCs in a given patient can express several “myeloma markers”, the MMC count was determined using the marker providing the maximum MMC count. To define that MMCs were detectable (*i.e.* positive MRD), a minimum of 100 MMCs events acquired was required.[5] Based on the counts of nucleated cells, which can be collected and processed by flow cytometer, the minimal detectable MMC count was 10^−5^ in the bone marrow or peripheral blood one day before HDM and 3 months post HDM, in line with previous reports.[4, 5] It was 2 × 10^−4^ seven days after HDM and ASCT due to the low leukocyte counts shortly after HDM. An additional labeling was performed with anti-CD34 and anti-CD45. CD45^+^ cells were first gated, HSCs were defined as [CD45^+^ and CD34^+^] cells, and the frequency of HSC was evaluated. Data were analyzed with FlowJo 9.1 software (Tree star, Ashland, Oregon). For leukapheresis product, the count of CD34^+^ HSCs was also measured using a FC500 (Beckman Coulter) flow cytometer.

### Culture of primary myeloma cells

Erythrocyte-lysed BM samples (the day before HDM and 7 days after HDM+ASCT) were cultured for 6 days at 4 × 10^5^ nucleated cells/mL in RPMI 1640 culture medium supplemented with 10% fetal calf serum (Invitrogen) and rIL-6 (2 ng/mL) (AbCys SA). At the end of the culture, cell count and viability were assayed using trypan blue exclusion. MMCs and N-PCs were quantified using flow cytometry as described above and apoptotic cells detected using Annexin V-FITC / 7AAD (Beckman Coulter, Fullerton, CA). Data were analyzed with Kaluza 1.1 software (Beckman Coulter, Fullerton, CA).

### Assay for cell communication signals

The concentrations of 30 cytokines / chemokines were measured in duplicates using multiplex bead based Luminex® technology (Invitrogen, Carlsbad, CA), with the following sensitivity: Interleukin-1β (IL-1β) (>15 pg/mL), IL-1RA (>30 pg/mL), IL-2 (>6 pg/mL), IL-2R (>47 pg/mL), IL-4 (>5 pg/mL), IL-5 (>3 pg/mL), IL-6 (>3 pg/mL), IL-7 (>10 pg/mL), IL-8 (>3 pg/mL), IL-10 (>5 pg/mL), IL-12 (>5 pg/mL), IL-13 (>5 pg/mL), IL-15 (>30 pg/mL), IL-17 (>16 pg/mL), Epidermal Growth Factor (EGF) (>20 pg/mL), Eotaxin (>5 pg/mL), Fibroblast Growth Factor-basic (FGF-b) (>22 pg/mL), Granulocyte-Macrophage Colony-Stimulating Factor (GM-CSF) (>5 pg/mL), G-CSF (>20 pg/mL), Hepatocyte Growth Factor (HGF) (>50 pg/mL), Interferon-α (IFN-α) (>20 pg/mL), IFN-γ (>5 pg/mL), Inducible Protein 10 (IP-10, CXCL10) (>5 pg/mL), Monocyte Chemotactic Protein-1 (MCP-1, CCL2) (>10 pg/mL), Monokine Induced by IFN-γ (MIG, CXCL9) (>45 pg/mL), Macrophage Inflammatory Protein 1α (MIP-1α, CCL3) (>10 pg/mL), MIP-1β (CCL4) (>10 pg/mL), Regulated on Activation Normally T-cell Expressed and Secreted (RANTES, CCL5) (>15 pg/mL), Tumor Necrosis Factor α (TNF-α)(>5 pg/mL), and Vascular Endothelial Growth Factor (VEGF) (>5 pg/mL). Measurements were performed on a Luminex® 100 analyzer (Luminex®, Austin, TX), and data were analyzed by a Luminex 100 IS® software version 2.3.

BAFF, APRIL, IGF-1, and IL-21 concentrations were assayed by enzyme-linked immunosorbent assay (ELISA). ELISA kits were purchased from R&D Systems for BAFF and IGF-1, from eBioscience for APRIL and from Abnova for IL-21. The sensitivity of the ELISA was 3.4 pg/mL for BAFF, 0.4 ng/mL for APRIL, 15.6 ng/mL for IGF-1 and 20 pg/mL for IL-21. Light absorbance was measured using a Mithras LB940 (Berthold technologies) runned by Mikrowin 2000® software.

### Statistical analysis

Statistical tests were performed using SigmaPlot software 11.0. Chi-square, unpaired or paired Wilcoxon signed rank test (when normality test failed) were used for group comparison. *P* values < .05 were considered to be associated with statistical significance.

## FUNDING

This work was supported by grants from ARC (SL220110603450, Paris France) and by Over-MyR FP7 EU grant. A.C. was supported by a fellowship from the Ligue Nationale Contre le Cancer.

## Supplementary Figures and Tables




