# Supplementary material for: Residual malignant and normal plasma cells shortly after high dose melphalan and stem cell transplantation. Highlight of a putative therapeutic window in Multiple Myeloma?
Source: Oncotarget. 2012 Oct 25;3(11):1335–47. doi: 10.18632/oncotarget.650 (PMC4539173; doi:10.18632/oncotarget.650)
Supplement: Supplementary file 1 [file oncotarget-03-1335-s001.pdf]

Residual malignant and normal plasma cells shortly after high dose melphalan and stem cell transplantation. Highlight of a putative therapeutic window in Multiple Myeloma? - caraux et al

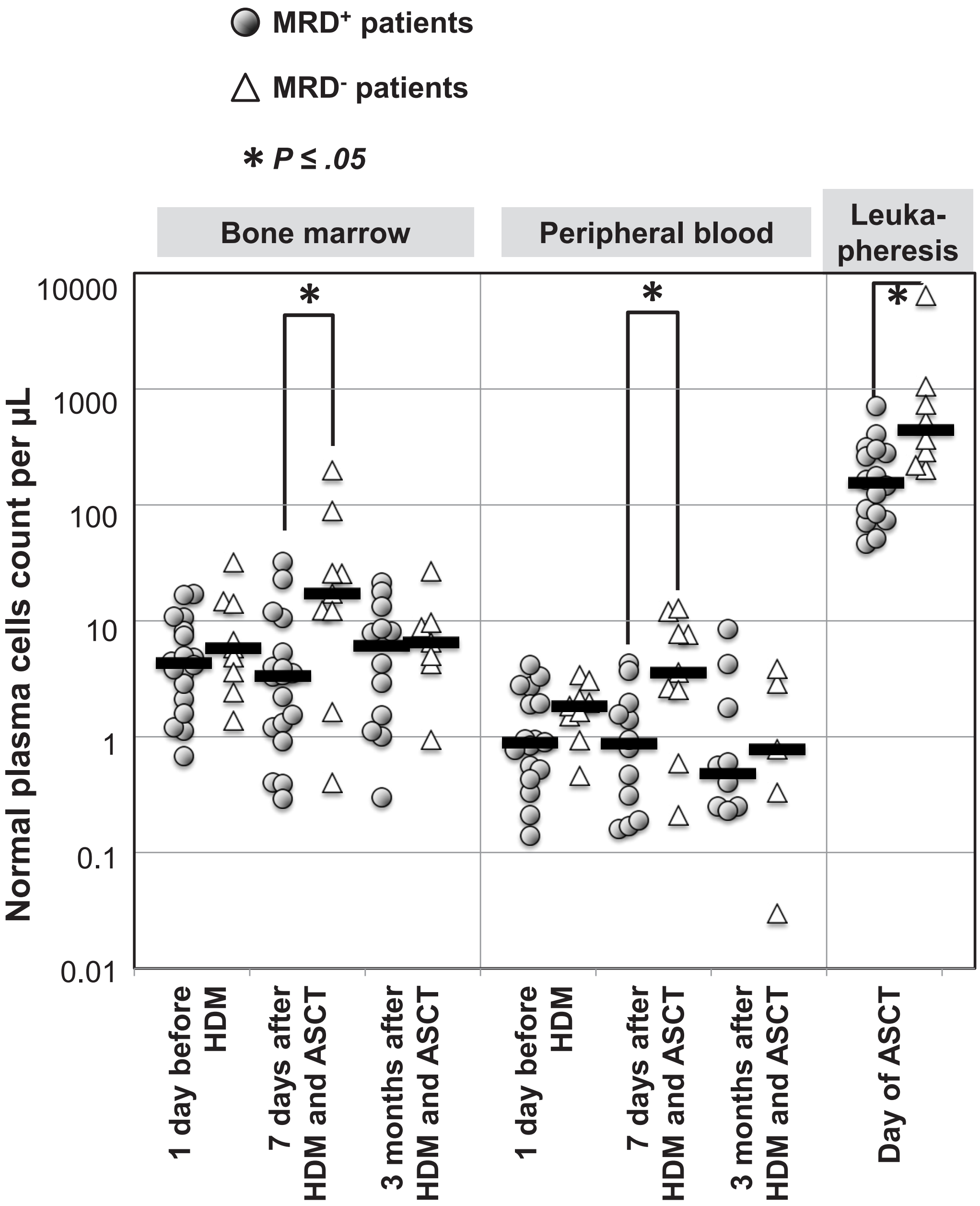

Supplemental Figure S1; Normal Plasma cell count before and after high dose melphalan in MRD+ patients compared to MRD- ones. Using 7 color-multiparameter flow cytometry, normal plasma cell (N-PC) count were assessed in bone marrow or peripheral blood samples of MRD^+ patients (black circle) compared to MRD^- ones (open triangle) after induction treatment (1 day before high dose melphalan, HDM), 7 days after HDM and autologous hematopoietic stem cell transplantation (ASCT), 3 months after HDM. N-PC count were also measured in the thawed stem cell leukapheresis product grafted to the patients. Data are the N-PC count for each of the MRD^+ patients (n=12) compared to MRD^- ones (n=5). The * indicates that the data in MRD^+ patients compared to MRD^- ones are significantly different ($P \leq 0.05$). 7 days after HDM+ASCT, the median N-PC count in the bone marrow and in the peripheral blood was higher in MRD^- patients compared to MRD^+ patients. And, the stem cell products collected from MRD^- patients and grafted to these patients contained 3 fold higher N-PC counts than those from MRD^+ patients.

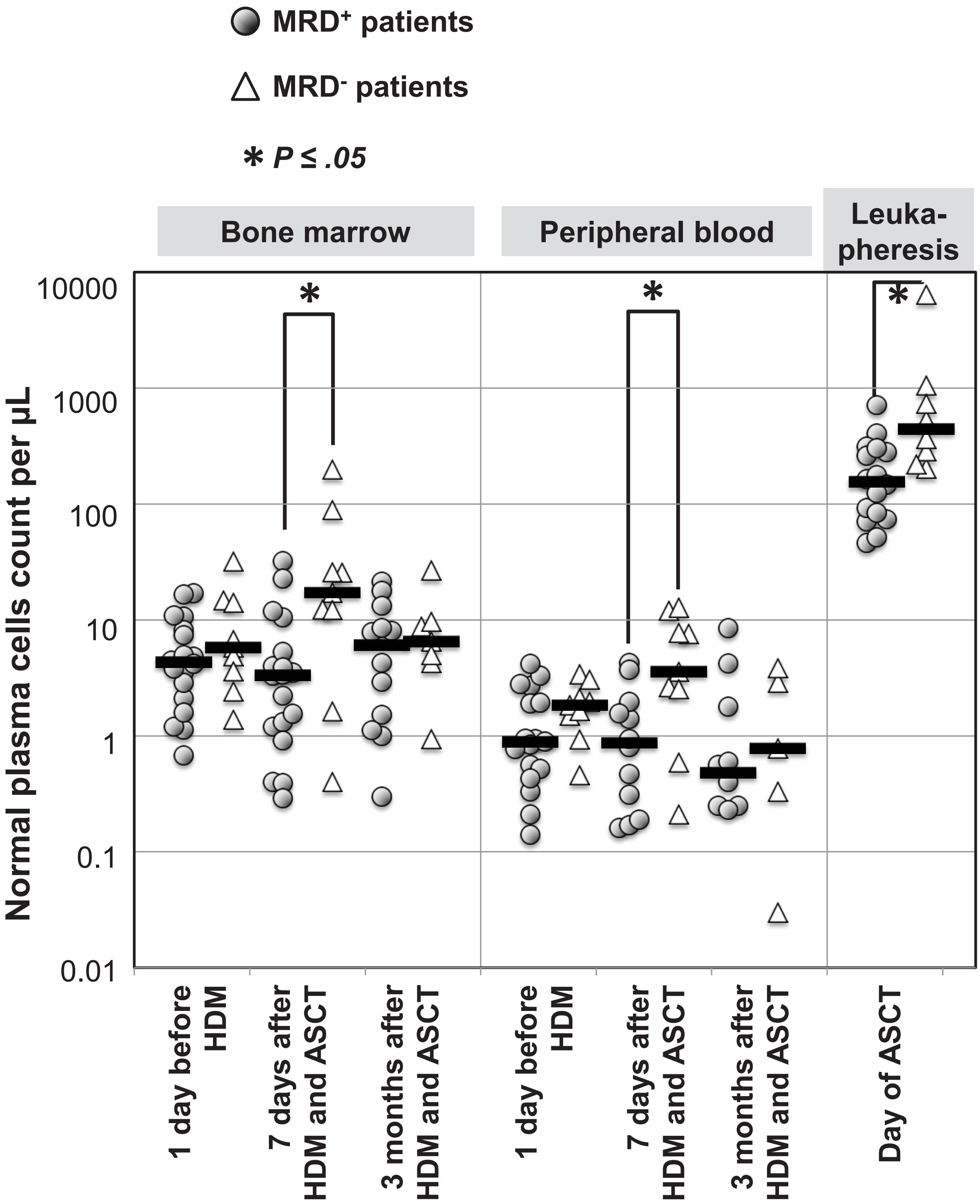

Supplemental Figure S2; Normal Plasma cell count before and after high dose melphalan in MRD+ patients compared to MRD- ones. Using 7 color-multiparameter flow cytometry, normal plasma cell (N-PC) count were assessed in the bone marrow or peripheral blood samples of MRD^+ (black circle) or MRD^- (open triangle) patients after induction treatment (1 day before high dose melphalan, HDM), 7 days after HDM and autologous hematopoietic stem cell transplantation (ASCT), 3 months after HDM. N-PC count were also measured in the thawed autologous leukapheresis product grafted to the patients. * indicates that the data in MRD^+ patients compared to MRD^- ones are significantly different ($P \leq .05$).
