# Supplementary material for: Residual malignant and normal plasma cells shortly after high dose melphalan and stem cell transplantation. Highlight of a putative therapeutic window in Multiple Myeloma?
Source: Oncotarget. 2012 Oct 25;3(11):1335–47. doi: 10.18632/oncotarget.650 (PMC4539173; doi:10.18632/oncotarget.650)
Supplement: Supplementary file 2 [file oncotarget-03-1335-s002.docx]

Residual malignant and normal plasma cells shortly after high dose melphalan and stem cell transplantation. Highlight of a putative therapeutic window in Multiple Myeloma? – Caraux et al

**Supplemental Table S1: In an given patient, multiple myeloma cells were identified on the basis of the monoclonal expression of Kappa or Lambda light chains together with the aberrant expression of one or several myeloma markers**

| **Patient** | **Lambda** | **Kappa** | **CD45** | **CD19** | **CD20** | **CD27** | **CD56** | **CD117** | **CD200** |
| --- | --- | --- | --- | --- | --- | --- | --- | --- | --- |
| **1** | **+** | **-** | **-** | **-** | **-** | **-** | **+** | **+** | **+** |
| **2** | **+** | **-** | **-** | **-** | **+** | **+** | **-** | **+** | **-** |
| **3** | **+** | **-** | **-** | **-** | **-** | **-** | **-** | **+** | **+** |
| **4** | **+** | **-** | **-** | **-** | **-** | **+** | **+** | **-** | **+** |
| **5** | **-** | **+** | **-** | **-** | **+** | **-** | **-** | **-** | **+** |
| **6** | **-** | **+** | **-** | **-** | **-** | **-** | **-** | **-** | **-** |
| **7** | **+** | **-** | **-** | **-** | **-** | **+** | **+** | **-** | **+** |
| **8** | **+** | **-** | **-** | **-** | **-** | **+** | **+** | **-** | **+** |
| **9** | **-** | **+** | **-** | **-** | **-** | **-** | **+** | **-** | **-** |
| **10** | **-** | **+** | **-** | **-** | **-** | **-** | **+** | **-** | **+** |
| **11** | **+** | **-** | **-** | **+** | **-** | **-** | **+** | **-** | **+** |
| **12** | **-** | **+** | **-** | **-** | **-** | **-** | **+** | **-** | **+** |
| **13** | **+** | **-** | **-** | **-** | **-** | **+** | **+** | **-** | **+** |
| **14** | **-** | **-** | **-** | **-** | **-** | **-** | **+** | **-** | **+** |
| **15** | **-** | **+** | **-** | **-** | **-** | **-** | **+** | **+** | **-** |
| **16** | **+** | **-** | **-** | **-** | **-** | **-** | **+** | **+** | **-** |
| **17** | **-** | **+** | **-** | **-** | **-** | **-** | **+** | **-** | **+** |
| **18** | **+** | **-** | **-** | **+** | **-** | **-** | **+** | **-** | **-** |
| **19** | **+** | **-** | **+** | **-** | **+** | **+** | **-** | **-** | **+** |
| **20** | **-** | **+** | **-** | **-** | **-** | **+** | **+** | **+** | **+** |
| **21** | **+** | **-** | **-** | **-** | **+** | **-** | **-** | **-** | **+** |
| **22** | **-** | **+** | **-** | **-** | **-** | **-** | **+** | **-** | **+** |
| **23** | **+** | **-** | **+** | **-** | **-** | **+** | **-** | **-** | **+** |
| **24** | **+** | **-** | **+** | **-** | **+** | **+** | **-** | **-** | **+** |
| **25** | **-** | **+** | **-** | **-** | **-** | **-** | **-** | **-** | **-** |
| **26** | **-** | **+** | **-** | **-** | **-** | **-** | **+** | **-** | **+** |
| **27** | **+** | **-** | **-** | **-** | **-** | **+** | **+** | **-** | **+** |

*CD20, CD56, CD117, CD200 were positive if more than 20% of the plasma cells displayed a level of expression. CD45, CD19, CD27 were negative if more than 20% of the plasma cells displayed an absence of expression. In grey, myeloma markers used to identify multiple myeloma cells (MMCs).*
